# Pennsylvania State Dental Society

**Published:** 1891-07

**Authors:** 


### PENNSYLVANIA STATE DENTAL SOCIETY.

(Continued from page 407.)

### DISCUSSION OF DR. FAUGHT'S PAPER ON "THE RELATION BETWEEN DENTAL CARIES AND DIET."

(FOR Dr. Faught's paper, see page 448.)

Dr. Leffmann said he has been for some years a convert to the theory of micro-organisms; he has come to the conclusion that bacilli are the origin of the destructive process known as caries of the teeth. Nevertheless, the possible effect of constitutional causes, of nervous derangements must not be lost sight of.

Micro-organisms produce chemical compounds which, doubtless, exert a local deleterious influence on the teeth, but they are perhaps not wholly to blame. As to diet, he cited the fact that while an article of food may be high in nitrogen, it need not necessarily, on that account, be the most nutritious, because the nitrogen may not be given up for proper assimilation by the digestive apparatus. Mushroom and oatmeal were given as examples, the former being highly nitrogenous, yet generally acknowledged to be lacking in nutritious qualities, while others less nitrogenous are more beneficial.

Dr. Gerhart said while he has no fault to find with the theories advanced by both the essayist and Dr. Leffmann, yet, on one point, he must take exception to them, and that is that micro-organisms are responsible for the effect of decay of the teeth. All things are the results of certain laws. Our functions are due to, and developed by, our environments; and when the conditions to which we have become adapted are deranged, our systems will suffer accordingly. When the teeth perform their intended functions they will resist the influences which tend to their destruction. This point the speaker illustrated by reference to certain tribes of the Sandwich Islands, who formed an exception to the rule of savages generally, in that, instead of eating their food in a raw state they cooked it. Consequently, as an examination of their skulls showed, their teeth decayed, and were attacked by all the diseases which those of civilized nations suffer, while neighboring tribes, who know not the art of cooking, seem to be exempt from diseases of their dental organs.

Dr. C. S. Beck said he does not believe in the direct influence of modes of life. An examination of ancient skulls in the Academy of Natural Sciences of Philadelphia will show teeth as variable in states of preservation and decay as those of to-day. Evidences are found in these old specimens of the existence of all the diseases to which teeth of our age are subject, such as decay, alveolar abscess, abrasion, erosion, etc. He believes oatmeal to be rather injurious than beneficial to the teeth by its effect upon the system. The manner of taking our food rather than the kind of food we eat he believes to be responsible for the poor condition of our teeth. As a nation we are too busy, and do not devote sufficient time to eating. In support of the claim that Americans have poorer teeth than representatives of other civilized nations, the speaker cited the alleged fact that the latter often have good teeth until they come to live among us, when their teeth commence to deteriorate. This, he said, he has seen illustrated in the cases of Irish servant girls.

Dr. Joseph R. C. Ward said his experience is that representatives of other countries, notably the Irish, whom Dr. Beck referred to as having good teeth, have no better teeth than Americans. The case of his own servant and others bear him out in this conclusion. He also spoke of the corrective influence of acid food in the extensive accumulation of lime-salts upon the teeth.

Dr. Beck asked Dr. Leffmann for his opinion as to the influence of oatmeal and other cereals upon the teeth. Dr. Leffmann, replying to Dr. Beck, said that, in his opinion, oatmeal and like cereals are overrated as vehicles for supplying the system with needed lime-salts. People who use them habitually are often found to have poor teeth. In support of the oatmeal theory, the Scotch people, who use it largely as an article of diet, are often held up as giving evidence, by the soundness of their teeth, of its value in this direction. But in answer to this argument the fact might be urged that they are also liberal consumers of spirituous liquors. Would it be any less logical, then, to attribute the preservation of their dental apparatus to the use of whiskey?

Dr. Fordham, in speaking of the supposed influence of oatmeal, cited the case of his own family, who, by his direction, have always used oatmeal, and yet the teeth of his own children, six in number, are more carious than those of any other of his patients of the same ages.

Dr. L. Jack asked Dr. Leffmann as to the possible eventual neutralizing effects of slight alkaline salivary conditions, and, on the other hand, of the possible promotive influence in the production of bacteria of a slightly acid condition of the saliva, and then referred to the deleterious influence in the promotion of caries in the cases of descendants of native Indians after their admixture by marriage with Caucasians.

Dr. Leffmann said that bacteria produced acid up to a certain point, at which the acid they produce will result in their own destruction. He also further explained the germ theory.

Dr. Beck cited the case of a patient, a boy sixteen years of age, of an extremely nervous temperament, and whose teeth are so persistently carious that no treatment has any effect. Constitutional treatment and travel are alike futile. His nervous condition is such that the least possible excitement creates intense disturbance and shock.

Dr. Leffmann, speaking of nervous impressions, related the case of a mother who was so much shocked by seeing a window-sash fall upon her child's hand that shortly afterwards a very perceptible irritation appeared on her own hand,—swelling and discoloring to correspond with the injury received by the child.

On motion, subject passed.

DISCUSSION OF DR. EISENBREY'S PAPER ON "IRREGULARITIES."

(For Dr. Eisenbrey's paper, see page 444.)

Dr. A. Boice illustrated on a black-board the application of a gold band in the treatment of irregularities, and urged the importance of using silk ligatures applied dry and tightly, as recommended by the essayist.

Dr. C. S. Beck exhibited models of a case spoken of before the Society a year or so ago. He brings these models to the Society's notice, he said, to show, by the results, the folly of practitioners both in diagnosis and prognosis, and then explained his method of procedure with bands, ligatures, and jack-screws in the case illustrated.

Dr. J. A. Libbey said that he was not present at the meeting referred to by Dr. Beck, but thinks the models exhibited show that the diagnosis was correct. He called attention to the danger of beginning treatment too early, and related a personal experience in bringing into place the lateral incisors of a young subject, in which the result was absorption of the roots, necessitating, of course, their extraction.

Dr. L. Jack, speaking of the too early treatment for irregularities, said that great harm may result by the premature closure of the apical foramen.

Dr. H. E. Roberts spoke of the difficulty experienced in inducing children to submit to treatment before they are old enough to appreciate the end sought.

Dr. W. H. Fundenberg considered it a very difficult matter to express an opinion from the mere observation of models. It is necessary in most cases to see the patient in order to get a full understanding of the case. The objection to Dr. Eisenbrey's ligatures is the endurance required in patients of tender years in the adjustment and wearing of such delicate appliances. The doctor cited a case in practice illustrating his method of procedure, which is opposed to ligatures, gold-bands, etc., that cannot be removed by the patient for the purpose of cleansing.

Dr. Ward pleads guilty to the charge of extracting teeth in regulating, and does not consider it a heinous offence either; believes it better in cases of extreme malposition to extract than to force such teeth into already crowded arches, and differs with the essayist, Dr. Beck, and others in the length of time required. He cannot conceive of a case requiring as much as a year to complete it.

Subject passed.

